# The impact of BMI on breast cancer – an updated systematic review and meta-analysis

**DOI:** 10.1097/MD.0000000000036831

**Published:** 2023-02-02

**Authors:** Nikolaos Tzenios, Mary E. Tazanios, Mohamed Chahine

**Affiliations:** a Public Health and Medical Research, Charisma University, Grace Bay, Turks and Caicos Islands, Train to Teach in Medicine, Department of Postgraduate Medical Education, Harvard Medical School, MCPHS University, Boston, MA; b Clinical Research, TRG GEN+, Beirut, Lebanon; c Biological and Chemical Technology, International Medical Institute, Kursk State Medical University, Kursk, Russian Federation.

**Keywords:** breast cancer, breast neoplasm, obesity, overweight, risk factor

## Abstract

**Background:**

Breast cancer is the most frequent form of cancer in women all over the world. It is the main cause of cancer death and the most often diagnosed cancer in women in 140 of the world’s 184 countries. The link between breast cancer risk and body mass index (BMI) has gotten increasing attention in recent years, although the results are still debatable. Therefore, the current systematic review and meta-analysis evaluate the impact of BMI on breast cancer.

**Methods:**

The current study was carried out as a systematic review and meta-analysis, following the Preferred Reporting Items for Systematic Reviews and Meta-Analyses (PRISMA) guidelines. We systematically searched Cochrane, Google Scholar, PubMed, EMBASE and Scopus databases to identify eligible articles impact of BMI on breast cancer with the appropriate Medical Subject Headings (MeSH). The Newcastle–Ottawa checklist was used for the risk of assessment for the included studies. Meta-analysis was performed using Review Manager 5.3 software.

**Results:**

Forty-six studies were included in the current review, which met the selection criteria of the current review. Among included 46 studies in this review, 50% (n = 23) of the studies found the HER2 type of breast cancer followed by triple-negative and HR-positive. The obesity was significantly higher in the case group compared with the control group (*P* < .001). Heterogeneity between the 14 studies is medium (*I*^2^ = 72%). In this review, there was no significant relation between overweight and breast cancer in women (*P* > .05). Heterogenecity between the 14 studies is medium (*I*^2^ = 89%). However, after removing the publication bias a significant relation between overweightness and breast cancer in women (*P* = .0005) was observed.

**Conclusion:**

Obese breast cancer patients are a specific type of patient. They are more likely to develop cancer. Their need to surgery and radiation may cause greater difficulties. Obesity and overweight in women greatly increase the risk of breast cancer, according to the findings of the current meta-analysis. To confirm these findings and understand the pathogenic pathways, more research is required.

## 1. Introduction

Cancer is one of the world’s major causes of death. The most frequent cancer among women is breast cancer and is one of the most important causes of death.^[1,2]^ In 2012, the International Agency for Research on Cancer reported 1.7 million breast cancer new cases, with 6.3 million new patients diagnosed in the previous 5 years.^[3]^ Every year, almost 1.5 million women worldwide are diagnosed with breast cancer.^[4]^ Breast cancer is a type of metastatic cancer that can spread to other parts of the body, including the bones, lungs, liver, and brain, making it uncurable. A good prognosis and a high survival percentage can be achieved if the cancer is detected early.^[5]^ The higher incidence of breast cancer in epidemiological studies among a group of women actively involved in social and professional life highlights the need for multidirectional investigations to identify risk factors linked to the development of this form of cancer.^[6]^ Breast cancer may be caused by a number of environmental, genetic, and lifestyle factors. Alcohol consumption, obesity, lack of physical activity, and overweight has been linked to 21% of all breast cancer deaths worldwide.^[7]^

Obesity, defined as a body mass index more than 30, is a chronic condition with a rising global prevalence that contributes to major health problems in most countries.^[8]^ Around 40% of the world’s female population is overweight (BMI of 25 kg/m^2^), according to the World Health Organization, and 15% is obese (BMI of 30 kg/m^2^). These numbers continue to increase.^[9]^ Overweight, lack of physical activity, and obesity have been identified as the major risk factors in high and middle-income nations, accounting for 18%.^[3]^ Multiple research relates it to the fact that adipocytes cause chronic inflammation in obesity leading to a rise in the systemic levels of cytokines as interleukin 6 and tumor necrosis factor.^[10]^ Obesity is a known risk factor for metabolic syndrome, type 2 diabetes, and heart attacks^[11]^; the latter is still the leading cause of death in women with early-stage breast cancer.^[12]^ Obesity’s strong impacts on breast cancer are caused by a variety of factors. Moreover, high BMI level is associated with a risk of several cancers, as well as an elevated cancer mortality.^[13]^ Obesity is widely recognized as a poor prognostic factor for breast cancer, despite being shown as a risk factor.^[14,15]^ With increasing BMI, the incidence of HR-positive breast cancer rose and HR-negative did not change. While the false-negative rate was similar over a wide range of BMIs, obese women had more cancers found on screening and at a later stage.^[16]^ In a review by Zahmatkesh et al,^[3]^ estimate the odds ratio of obesity and overweight as risk factors for breast cancer. Obesity, as well as overweight, was shown to have a strong link to the breast cancer risk. Recently, Harborg et al,^[17]^ have evaluated the correlation between obesity and the outcome in triple-negative breast cancer (TNBC) patients. According to the review findings, being overweight is linked to a shorter disease-free and overall survival in TNBC patients. However, the relationship between BMI and the risk of breast cancer has received much interest, but the findings are still up for discussion.^[18,19]^ Therefore, the current systematic review and meta-analysis evaluating the impact of BMI on breast cancer is lead.

## 2. Materials and methods

### 2.1. Study design

The PRISMA guidelines was followed to conducting this systematic review and meta-analysis.^[20]^

### 2.2. Search strategy

We conducted a manual and electronic search and identified literature published up to November 30, 2021. Literature searches were carried out on databases such as Cochrane, Google Scholar, PubMed, EMBASE and Scopus with the appropriate MeSH and phrases. Different types of keywords were used for the search strategies such as “body mass index,” OR “BMI,” OR “obesity,” OR “overweight,” OR “weight,” OR “adiposity” OR “weight change” AND “Cancer, breast,” OR “breast cancer,” OR “breast carcinoma” OR Carcinoma, human mammary OR human mammary neoplasm. The bibliographic sources of the selected articles were also screened.

### 2.3. Inclusion and exclusion criteria

All the published studies that report the impact of BMI on breast cancer, calculating BMI at the start and then monitoring the incidence of breast cancer during follow-up, original research articles, articles published in English were included in this review. Only studies having 95% confidence intervals (CI) on odds ratios (OR) among newly diagnosed or histopathologically confirmed breast cancer patients who had not previously had radiation, surgery, or chemotherapy were included.

Studies that evaluated other than breast cancer, assessing the impact of other comorbidities in breast cancer patients other than BMI, recurrences risk estimates of breast cancer, gray literature, including presented abstracts, letters to the editors, commentaries, systematic review, narrative reviews or meta-analysis articles and unavailability of the full text of the article were excluded from the current review.

### 2.4. Article screening

Relevant articles were chosen for full-text screening after applying the eligibility criteria. Two authors have independently performed articles screening process and eligibility assessment. In case of some contradictions between the authors, the decision was made by an unbiased third party. The articles were initially screened based on their title, followed by the article’s abstract. The case title and abstract of the articles were irrelevant to the present investigation; these were excluded from the secondary screening.

### 2.5. Data extraction

The followed data was extracted from the selected articles, including the first author’s name, country, study design, sample size, age, type of breast cancer, height, weight, stages, test methods, menopausal status, treatment and follow-up duration were extracted from the selected article.

### 2.6. Risk of bias assessment

Two reviewers independently assessed the quality assessment for the selected studies using Newcastle–Ottawa checklist. A discussion with the third reviewer solved divergences. The following are some of the Newcastle-Ottawa checklist criteria that will be used to assess study quality: Methods: (setting, participants, variables, data sources/measurement, bias), Results: (main results), Discussion: (limitations, generalizability). The total score ranged from 0 to 9, while scores less than 3, less than 6 and between 7 and 9 will be considered as low, moderate and high-quality studies, respectively.

### 2.7. Statistical analysis

Review Manager 5.3 was used to conduct the meta-analysis (The Cochrane Collaboration, 2014). The combined effect size was calculated using a meta-analysis with a 95% CI. Pooled OR and 95% CI estimates was computed using random-effects modeling and forest plots were used to display the results. Random effects modeling was used because, regardless of the level of statistical heterogeneity. The *I*^2^ statistic was used to analyze the clinical heterogeneity of included studies using the Cochran Q test. Significant heterogeneity was defined as *I*^2^ larger than 50%. To identify the source of heterogeneity, sensitivity analysis was performed by assessing the influence of different study features such as sample size, publication year, and menopausal status. Visual inspection of a funnel plot was used to determine publication bias. The statistical tests were two-sided, and *P* < .05 was used to determine statistical significance.

### 2.8. Ethical considerations

No ethical approval or patient consent was required in this review because all analyses was based on already published studies. To prevent ethical issues with regards to plagiarism and copyrights, the findings from the selected articles were duly paraphrased along with acknowledging the work of the authors via the addition of references.

## 3. Results

### 3.1. Eligible studies

**Figure 1.**
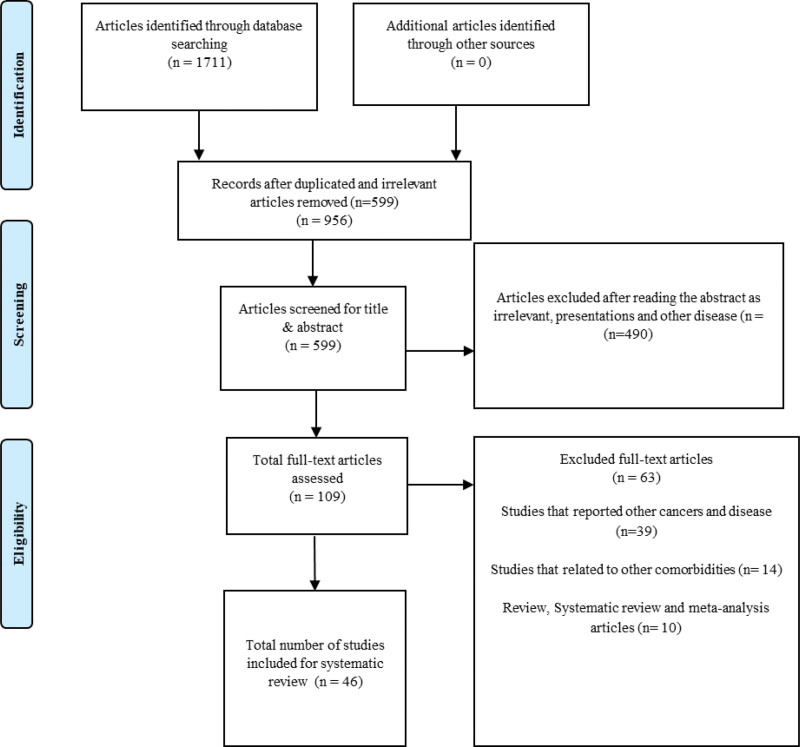
PRISMA chart.

A total of 1711 articles were found in searched databases, including Cochrane, Google Scholar, PubMed, EMBASE and Scopus, of which 1112 were initially eliminated owing to repetition and irrelevance. After analyzing the titles and abstracts at the first screening level, 490 articles were further removed. For full-text evaluations, a total of 109 potential relevant articles were chosen, of which 63 articles were further excluded as studies that reported other cancers and disease (n = 39), studies that related to other comorbidities (n = 14) and review, systematic review and meta-analysis articles (n = 10). Finally, 46 studies that met the criteria for inclusion in the systematic review, as detailed in the PRISMA flow chart (Fig. 1), were included in this review.

### 3.2. Baseline characteristics study

Table 1 shows the baseline characteristics of the studies that were included. Out of 46 included studies, 9 studies were published in Iran,^[51–56,58,62,63]^ 4 studies published in China,^[23,30,31,50]^ 4 studies published in Italy,^[24,40,42,49]^ 4 studies published in Japan,^[25,32,36,38]^ 4 studies published in USA,^[33,39,45,46]^ 4 studies published in Thailand,^[59,61,64,65]^ 2 studies published in Korea,^[27,34]^ 2 studies published in Germany,^[35,43]^ 2 studies published in Malaysia,^[57,66]^ whereas remaining 1 studies published in different countries including UK,^[21]^ France,^[22]^ Jordan,^[26]^ North Africa,^[28]^ California,^[29]^ Australia,^[37]^ Turkey,^[41]^ UAE,^[44]^ Mexico,^[47]^ New york^[48]^ and Singapore.^[60]^ In addition, the majority of included studies were case-control study design (n = 16) followed by observational (n = 12) and retrospective studies (n = 8). A total of 2,95,260 patients were included in the study, with sample sizes ranging from 115 to 47,271. All the included patients in this review were female.

**Table 1:** Characteristics of included studies.

S.No	Author and year	Country	Study design	Sample size/Gender	Age (years) Range/Mean/Median	Type of breast cancer	Height	Weight		Stages	Test methods	BMI range	Physical activity (PA) assessment details	Side effects	Adjustment factors	Menopausal status	Treatment	Follow up duration	Main findings
								Premenopausal women	Post menopausal women										
1	Smith et al,^[21]^	UK	Observational study	N = 10,653/Female	Range = 35–70	NR	NR	NR	Under weight = 50.5% Overweight = 57.9% Obese = 54.9 %	NR	Kaplan–Meier analysis	95% confidence interval (CI), The range of BMI is < 25 kg/m^2^ to ≥ 30 kg/m^2^	NR	NR	Age, hormone replacement therapy (HRT) use, current or previous history of smoking and baseline menopausal status, preventive therapy	Postmenopausal	Proportional hazards regression model	16.2 yr	Higher BMI is associated with greater breast cancer risk in postmenopausal women at increased risk of the disease, but no effect was observed in premenopausal women
2	Saleh et al,^[22]^	France	Retrospective cohort study	N = 22,463/Female	Range = 53–60	HR + HER2-HER2 + TNBC	NR	Underweight = 267 (42%) Normal weight = 2694 (45%) Overweight = 1843 (50%) Obese = 1386 (53%)	Underweight = 247 (39%) Normal weight = 2103 (35%) Overweight = 1302 (35%) Obese = 850 (32%)	NR	Chi-square tests or ANOVA tests, Kaplan–Meier analysis	95% confidence interval (CI), The range of BMI is < 18.5 kg/m^2^ to ≥ 30 kg/m^2^ Underweight = 17.5 (16.7–18) Normal = 22.2 (20.8–23.6) Overweight = 27.1 (26.0–28.3) Obese = 33.3 (31.2–36.4)	NR	NR	Chemotherapy, Endocrine therapy	Premenopausal, Postmenopausal	Cox proportional hazards model	48.6 mo	Overweight and obesity are not associated with poorer outcomes in women with metastatic disease, while underweight appears as an independent adverse prognostic factor
3	Abubakar et al,^[23]^	China	Cohort study	N = 8179/Female	Mean = 51.8 (<35–>55)	Luminal A-like, luminal B-like, HER2-enriched, and triple negative breast cancer [TNBC]	NR	NR	NR	NR	Chi-square (ΔLRχ^2^) tests	95% confidence interval (CI) The range of BMI is < 18.5 kg/m^2^ to > 30 kg/m^2^ Underweight = 155 (2.1) Normal = 3946 (54.2) Overweight = 2586 (35.5) Obese = 595 (8.2)	Associations between breast cancer risk factors and tumor-related prognostic indicators were assessed in polytomous logistic regression models with quartiles of NPI and IHC4 score as outcomes and breast cancer risk factors as predictors.	breast cancer risk factors, including age, BMI, family history of breast cancer (FHBC)	The contribution of individual factors to model prediction was determined by assessing change in likelihood ratio Chi-square (LRχ2) when the factor was removed from the fully adjusted model.	Premenopausal, Postmenopausal	NR	NR	Elevated BMI was associated with low IHC4 score, connoting clinically less aggressive HR + tumors, NPI-related findings were supportive of poor prognosis among overweight/obese patients irrespective of tumor HR status.
4	Krasniqi et al,^[24]^	Italy	Observational study	N = 709/Female	Median = 54 (26–87)	HER2 + metastatic breast cancer (mBC)	NR	NR	NR	NR	Kaplan–Meier curves and relative log-rank tests	95% confidence interval (CI) The range of BMI is < 18.5 kg/m^2^ to > 30 kg/m^2^ Underweight/normal = 195 (100.0%) Overweight = 103 (100.0%) Obese = 61 (100.0%)	The overall survival (OS) variation determined by BMI was assessed within PFS1 quartiles	NR	The effect of BMI on prognosis was also confirmed in multivariate analysis of OS for overall population	NR	Pertuzumab and/or trastuzumab emtansine (TDM1)	6 mo	According to PFS1 quartiles a higher percentage of patients with high BMI and low disease burden progressed within 6 months of therapy. The effect of BMI on prognosis was also confirmed in multivariate analysis of OS for overall population
5	Gondo et al,^[25]^	Japan	retrospective cohort study	N = 3223/Female	Median = 54 (range 2.7–88.6)	ER, PR and HER2 receptor status	NR	Underweight = 189 (59.4%) Normal weight = 1098 (48.7%) Overweight = 163(29.9%) Obese = 42 (40.8%)	Underweight = 129 (40.6%) Normal weight = 1159 (51.4%) Overweight = 382(70.1%) Obese = 61 (59.2%)	stage I–IIIC invasive carcinoma stage I = 1772 (54.98%) stage II = 1243 (38.57 %) stage III = 208 (6.45 %)	Kaplan–Meier method and log-lank test	95% confidence interval. The range of BMI is < 18.5 kg/m^2^ to > 30 kg/m^2^ Underweight = 318 (9.9%) Normal = 2257 (70.0%) Overweight = 545 (16.9%) Obese = 103 (3.2%)	Tumour size and the number of lymph node metastases in patients who received neoadjuvant therapy were assessed before the treatment.	Obesity may be more influential in patients who can achieve higher therapeutic effects by hormonal therapy.	It was found that BMI was also a poor prognostic factor as well as tumor size, lymph node metastasis number, histological grade, which are known as prognostic factors	Premenopausal, Postmenopausal	ATAC is a randomized phase-III trial comparing adjuvant anastrozole to tamoxifen in postoperative treatment	57 mo	Patients without lymph node metastasis had no prognostic difference due to BMI, but in the lymph node-positive patient group the prognosis was statistically superior in the obese group
6	Ayoub et al,^[26]^	Jordan	retrospective study	N = 348/Female	Mean = 50.98 ± 10.96 (ranging from 24 to 83)	HER2 status, luminal A, luminal B, HER2-positive	NR	Normal weight = 43 (67.2%) Overweight = 52(44.1%) Obese = 58 (34.9%)	Normal weight = 21 (32.8%) Overweight = 66(55.9%) Obese = 108(65.1%)	Stage–I = 18 (5.2%) Stage–II = 170 (48.9%) Stage–III = 101 (29.0%) Stage–IV = 59 (17.0%)	Independent sample t-test analysis	The range of BMI is 29.52 ± 5.32 kg/m^2^ Underweight/normal = 64 (18.4) Overweight = 118 (33.9) Obese = 166 (47.7)	Multivariate logistic stepwise regression analysis was conducted to identify predictors for breast cancer recurrence and death. Pearson’s correlation analysis revealed a significant positive correlation between patient age and BMI at diagnosis	Jordanian obese breast cancer patients are at greater risk of breast cancer recurrence and reduced survival compared to their nonobese counterparts.	Stage and BMI were found by multivariate logistic regression as the only predictors for death in breast cancer patients. Advanced stage at diagnosis is a significant risk factor predictive for death among patients in multivariate regression	Premenopausal, Postmenopausal	NR	NR	Obesity was associated with advanced stage and grade of breast carcinoma at diagnosis. The impact of BMI on clinicopathologic characteristics and prognosis was confined to postmenopausal cases.
7	Cho et al,^[27]^	Korea	Observational study	N = 5668/Female	Median = 48 and 52	HR+/Her2-	NR	NR	NR	pT stage pT1 = 2581 (62.9%) pT2 = 1326 (32.3%) pT3 = 184 (4.5%) pT4 = 12 (0.3%)	Kaplan–Meier methods, Mann-Whitney test, chi-square test	95% confidence interval (CI), The range of BMI is < 25 kg/m^2^ to ≥ 25 kg/m^2^ Underweight/normal = 4103 (72.4%) Overweight = 1565 (27.6%)	NR	NR	NR	NR	Chemotherapy, Radiation therapy	NR	obesity was a poor prognostic factor for DFS and OS particularly in hormone receptor positive and Her2 negative breast cancer, while obesity was not a significant factor in other subtypes in Asian women
8	Al Jarroudi et al, m^[28]^	North africa	retrospective cohort study	N = 115/Female	Median = 47.1	Triple-negative breast cancer	NR	Underweight = 12 (35.3 %) Overweight = 73 (90.2 %)	Underweight = 22 (64.7 %) Overweight = 8 (9.8)	NR	chi-square test.	95% confidence interval (CI), The range of BMI is < 25 kg/m^2^ to ≥ 25 kg/m^2^ Underweight/normal = 34 (28.7%) Overweight = 82 (71.3%)	NR	NR	NR	Premenopausal, Postmenopausal	Adjuvant therapy by multivariate Cox proportional hazards models.	NR	Indicated that menopausal status may be a mitigating factor, with overweight premenopausal women at greater risk of death and progression than women with a normal weight.
9	Engmann et al,^[29]^	California	cohort study	N = 18 437/Female	Mean = 46.3 (3.7) to 61.7 (7.2)	NR	NR	Under weight = 0.93 (0.76–1.15) Overweight = 0.99 (0.93–1.07) Obese = 1.05 (0.94–1.18)	Under weight = 0.79 (0.67–0.93) Overweight = 1.23 (1.17–1.28) Obese = 1.54 (1.45–1.64)	NR	NR	95% confidence interval (CI). The range of BMI is < 18.5 to ≥ 35.0	easily assessed risk factors should be incorporated into risk prediction models to stratify breast cancer risk and promote risk-based screening and targeted prevention efforts.	Family history of breast cancer, history of benign breast biopsy, nulliparous	Risk factors selected a priori for analysis were dense breasts (heterogeneously dense or extremely dense), first-degree relative with breast cancer, history of benign breast biopsy, and nulliparity or age at first birth 30 years or older.	Premenopausal, Postmenopausal	NR	NR	The first estimate of PARP for clinical risk factors in pre-menopausal women, and suggest that with the exception of BMI, the PARP of most risk factors is similar among premenopausal and postmenopausal women
10	Bao et al,^[30]^	China	Observational cohort study	N = 518/Female	Mean = 53.4	HER2-positive	2.5 cm above the umbilicus and hip	NR	NR	Stage (%) I = 30.89 II = 55.60 III = 10.23	χ^2^ test and Fisher’s exact test	95% confidence interval (CI), The range of BMI is < 18.5 kg/m^2^ to ≥ 28 kg/m^2^ Underweight = 17 Normal = 253 Overweight = 183 Obese = 65	NR	NR	chemotherapy, and radiotherapy	Premenopausal, Postmenopausal	NR	9.1 yr	obesity pre-diagnosis and weight loss post-diagnosis was inversely associated with TNBC prognosis and maintained stable weight after cancer diagnosis for TNBC patients
11	Chen et al,^[31]^	China	Observational cohort study	N = 206/Female	Median = 48.5 years	HER2 receptor	NR	Underweight = 40 (49.4 %) Overweight = 73 (58.4 %)	Underweight = 41 (50.6 %) Overweight= 52(41.6 %)	NR	Kaplan–Meier methods, log-rank test, chi-square test	95% confidence interval (CI), The range of BMI is < 25 kg/m^2^ to ≥ 25 kg/m^2^ Underweight/normal = 81 (39.3 %) Overweight = 71 (34.5 %)	NR	NR	Adjuvant/neoadjuvant chemotherapy composed of anthracyclines and/or taxanes	Premenopausal, Postmenopausal	Chemotherapy, Radiation therapy	59 mo	Central obesity, especially with high BMI, was an independent prognostic factor for TNBC.
12	Kawai et al,^[32]^	Japan	Obervational cohort study	N = 20,090/Female	Mean = 57.3	luminal A, luminal B, HER2, triple negative, others	NR	Underweight = 696 (44.6 %) Normal weight = 3065 (44.9%) Overweight = 1923 (28.4 %) Obese = 222 (24.8%)	Underweight = 814 (52.2%) Normal weight = 3524(51.6%) Overweight = 4611(68.0%) Obese = 640 (71.4%)	Stage I = 8304 (41.3%) Stage II (IIA/IIB) = 9841 (49.0%) Stage III (IIIA/IIIB/IIIC) = 1945 (9.7%)	NR	95% confidence interval (CI), The range of BMI is < 18.5 kg/m^2^ to ≥ 30 kg/m^2^ Underweight = 1561 (138) Normal = 6833 (414) Overweight = 6784 (476) Obese = 4015 (298)	NR	NR	NR	Premenopausal, Postmenopausal	Chemotherapy, Endocrine therapy, Radiation therapy	6.7 yr	Being obese or underweight is associated with a higher risk of death among female breast cancer patients in Japan.
13	Cecchini et al,^[33]^	USA	A retrospective study	N = 15,538/Female	Mean = 50	HER2-positive	NR	NR	NR	NR	NR	The range of BMI is < 25.0 kg/m^2^ to ≥ 30 kg/m^2^	NR	NR	Cox proportional hazards regression to calculate adjusted hazards ratios (HR) for risk of death and recurrence	Premenopausal, Postmenopausal	Adjuvant treatment regimens: doxorubicin and cyclophosphamide followed by docetaxel	5.9 yr	The heterogeneity of breast cancer between different breast cancer populations and the different therapies used to treat them may modify any association that exists between BMI and breast cancer outcome
14	Jeon et al,^[34]^	Korea	Observational cohort study	N = 41,021/Female	Mean = 48 (range, 18 to 93)	HER2, Korean Breast Cancer Society	NR	Underweight BMI = 1.16(0.98–1.36) Normal BMI = 1.00 Overweight BMI = 1.26(1.18–1.35) Obese BMI = 1.51(1.32–1.72)		NR	chi-square test, Log-rank tests, Kaplan–Meier method	95% confidence interval (CI), The range of BMI is < 18.5 kg/m^2^ to ≥ 30 kg/m^2^ normal = 1387 underweight = 27,519 overweight = 10,483 obese = 1632	NR	NR	After adjusting for poor prognostic factors, such as tumor size, axillary lymph node metastasis, histologic grade, ER, PR and HER2 expression, a U-shaped association between BMI and mortality	NR	Adjuvant treatment (such as radiotherapy, chemotherapy, and hormonal therapy)	92 mo	U-shaped relationship between BMI at diagnosis and poor overall survival (OS) and breast cancer specific survival (BCSS) among all breast cancer patients, with the lowest risk observed among breast cancer patients with normal BMIs.Therefore, BMI at diagnosis and breast cancer subtype should be considered simultaneously in various treatment decision processes
15	Widschwendter et al,^[35]^	Germany	A retrospective study	N = 3754/Female	Median = 53 (21–86)	triple-negative breast cancer (TNBC), luminal B-like, Luminal A–like or HER2-positive	NR	Under weight = 932 (53.0 %) Overweight = 410 (33.9%) Obese = 153 (27.6 %)	Under weight = 826 (47.0 %) Overweight = 798 (66.1 %) Obese = 401 (72.4 %)	Nodal stage. pN0 = 1273 (33.9 %) pN1 1705 (45.4 %) pN2 511 (13.6 %) pN3 236 (6.3 %)	Kruskal-Wallis H test for age, the Mantel-Haenszel linear-by-linear association chi-square test	95% confidence interval (CI), The range of BMI is < 25.0 kg/m^2^ to ≥ 40 kg/m^2^ Underweight/normal weight = 1758 Overweight = 1208 Slightly obese = 554 Moderately obese = 177 Severely obese = 57	NR	NR	Luminal A-like breast cancer as revealed by univariate log-rank tests was confirmed by fully adjusted multivariate Cox regressions	Premenopausal, Postmenopausal	Adjuvant chemotherapy treatment with 3 cycles of epirubicin, fluorouracil and cyclophosphamide	65 mo	No significant impact of obesity on outcomes in the luminal A-like, luminal B-like and HER2-positive subtypes, but significantly poorer disease-free survival (DFS) and Overall survival (OS) in severely obese compared with underweight/normal weight patients with triple-negative breast cancer.
16	Ohara et al,^[36]^	Japan	Observational study	N = 184/Female	Median = 64	ER+/HER2-	NR	NR	NR	pT stage, n (%) T1 = 130 (70.7) T2 = 49 (26.6) T3 = 1 (0.5) T4 = 4 (2.2)	Kaplan–Meier methods, log-rank test, Fisher’s exact test, χ^2^ test	95% confidence interval (CI), The range of BMI is < 23.6 kg/m^2^ to ≥ 38.8 kg/m^2^ underweight = 120 overweight = 52 obese = 12	NR	NR	Endocrine therapy, (Anastrozole,Letrozole, Exemestane) adjuvant chemotherapy (trastuzumab)	Premenopausal	Anastrozole treatment	46.1 mo	PgR/BMI status may serve as a useful prognostic factor in postmenopausal women with ER + and HER2–breast cancer treated with adjuvant aromatase inhibitors
17	Robinson et al,^[37]^	Australia	Bupa cohort study	N = 1155/Female	Mean = 58.4 ± 11.6	HR+, HER2 − breast cancer	NR	NR	NR	Stage 1 = 88.9%	Kaplan–Meier methods, log-rank test, chi-square test	95% confidence interval (CI), The range of BMI is < 18.5 kg/m^2^ to ≥ 30 kg/m^2^ Underweight = 14 (1.2%) Normal = 528 (44.0%) Overweight = 374 (31.2%) Obese = 253 (21.1%) Morbidly obese = 30 (2.5%)	NR	NR	Oral adjuvant endocrine therapy (OAET)	NR	Chemotherapy, radiotherapy and oral adjuvant endocrine therapy (OAET)	5.6 yr	Moderate to severe obesity is associated with a poorer invasive breast cancer prognosis; this is also true for women with Stage 1 disease, and is independent of age and treatment.
18	Asaga et al,^[38]^	Japan	Observational study	N = 135/Female	Median = 54	Triple-negative breast cancer, HER2	NR	NR	NR	T stage T1 = 6 T2 = 75 T3 = 34 T4 = 20	Kaplan–Meier analysis	95% confidence interval (CI), The range of BMI is < 18.5 kg/m^2^ to ≥ 25 kg/m^2^ Underweight/normal = 10 Overweight = 97 Obese = 28	All patients received physical examinations every 3–6 months, and blood tests and chest x-rays at least annually as outpatients after surgical treatment	NR	Chemotherapy, and radiotherapy	Premenopausal, Postmenopausal	Cox proportional hazards modeling	49.2 mo	Pathologic complete response (pCR) is not an independent significant prognostic marker for TNBC patients receiving PST. Clinical response is a stronger surrogate marker than pCR for a favorable prognosis
19	Crozier et al,^[39]^	USA	International cohort study	N = 3017/Female	Median = 50 (50–59)	HER2-positive	NR	Under weight = 562 (64%) Overweight = 440 (52%) Obese = 608 (47 %)	Under weight = 315 (36%) Overweight = 402 (48%) Obese = 690 (53 %)	NR	Kaplan–Meier methods, log-rank test, chi-square test	95% confidence interval (CI), The range of BMI is < 25.0 kg/m^2^ to ≥ 30 kg/m^2^ Underweight/normal = 877 Overweight = 842 Obese = 1298	NR	NR	Cox proportional hazards analysis, stratified by hormone receptor status and lymph node status and adjusted for race and age	Premenopausal, Postmenopausal	adjuvant therapy, including trastuzumab.	5.3 yr	Patients with early stage, HER2-positive breast cancer and normal BMI had a better 5-year DFS compared with overweight and obese women. The current results indicated that adjuvant trastuzumab improves clinical outcome regardless of BMI
20	Mazzarella et al,^[40]^	Italy	Cohort study	N = 1250/Female	Median = 35–65	HER2+	NR	Under weight = 325 (58.9%) Overweight = 48 (32.4%) Obese = 13 (22 %)	Under weight = 227 (41.1%) Overweight = 100 (67.6%) Obese = 46 (78 %)	NR	Kaplan–Meier methods, log-rank test, chi-square test	95% confidence interval (CI), The range of BMI is < 18.5 kg/m^2^ to ≥ 30 kg/m^2^	NR	NR	Multivariable analyses confirmed the results observed in the univariate analysis	Premenopausal, Postmenopausal	Adjuvant therapy, including trastuzumab.	8.2 yr	Obesity significantly correlates with worse overall survival and cumulative incidence of distant metastases in ER/HER2 positive breast cancer
21	Turkoz et al,^[41]^	Turkey	Observational cohort study	N = 733/Female	Mean = 38.4 ± 6.7	Luminal, HER-2 overexpressing, Triple negative	NR	NR	NR	NR	Kaplan–Meier methods, log-rank test, chi-square test	95% confidence interval (CI), The range of BMI is < 18.5 kg/m^2^ to ≥ 30 kg/m^2^ Underweight/normal = 43.7% Overweight = 33.0% Obese = 23.3%	NR	NR	After adjustment for the prognostic factors (age, tumor size, nodal involvement, grade, lymphovascular and perineural invasion, extracapsular extension and hormonal status),	Premenopausal	NR	29 mo	Obesity is associated with estrogen (ER) and progesterone receptor (PR) negative tumors and poor overall (OS) in premenopausal women with breast cancer.
22	Biglia et al,^[42]^	Italy	Observational study	N = 2148/Female	Mean = 45 to 65	Luminal A, B, HER-2 and basal-like	NR	Underweight (BMI < 18.9) = 35 (6%) Normal weight (19 < BMI < 24.9) = 390 (65.8%) Overweight (25 < BM < 29.9) = 108 (18.2%) Obese (BMI > 30) = 59 (10%)	Underweight (BMI < 18.9) = 47 (3%) Normal weight (19 < BMI < 24.9) = 747 (48%) Overweight (25 < BM < 29.9) = 521 (33.5%) Obese (BMI > 30) = 241(15.5%)	NR	χ2 or Fisher Exact test, ANOVA test, Non-parametrical tests (Mann–Whitney *U* test and Wilcoxon test), Kaplan–Meier method	98% for women with BMI < 25 96% with BMI ≥ 25	Overweight/obese women had significantly larger tumor at diagnosis than under/normal weight women, both in premenopause and postmenopause	NR	At multivariate survival analysis BMI was not an independent prognostic factor.	Premenopausal, Postmenopausal	NR	60 mo	No statistically significant between breast cancer subtypes (luminal A, B, HER-2 and basal-like) and BMI in both pre and postmenopausal women.
23	Schmidt et al,^[43]^	Germany	Prospective cohort follow-up of case-control study	N = 3393/Female	Median = 62.7 (interquartile range: 57.6–66.7)	HER2-neu status	NR	NR	NR	Stage I–IIIa = 317 (10.9%) Prognosis, Stage IIIb or IIIc carcinomas, Stage IV at primary diagnosis (baseline)	χ2 test for nominal variables and Kruskal–Wallis test for ordinal variables.	73.9% normal adult BMI (18–25 kg/m^2^) Underweight = 833 Normal = 687 Overweight = 619 Obese = 579	Pre-diagnosis recreational PA since age 50 y until breast cancer diagnosis (median 12.7 y, range 7.6–16.7 y) was assessed using an interviewer-administered questionnaire	NR	prognostic factors was collected from clinical and pathological records.	Postmenopausal	Chemotherapy = 47.9% HT/Tamoxifen = 77.5%	5.6 yr	Breast cancer patients with a physically inactive lifestyle pre-diagnosis may decrease prematurely irrespective of their cancer prognosis. However, There were no interactions with other tumor characteristics, BMI or smoking.
24	Dawood et al,^[44]^	UAE	A retrospective study	N = 2311/Female	Median = 48 to 51	Triple receptor-negative breast cancer (TN)	NR	Under weight = 389(49.2%) Overweight = 288 (41.8%) Obese = 294 (35.9%)	Under weight = 402(50.8%) Overweight = 401(58.2%) Obese = 525 (64.1%)	Overall stage III –I = 5.19 (95% CI 3.93–6.85) Recurrence-Free stage III –I = 4.79(95% CI 3.72–6.17)	X2 test, Kaplan–Meier method	95% confidence interval (CI), The range of BMI is < 25 kg/m^2^ to 30 + kg/m^2^ Underweight = 794 over weight = 692 Obese = 825	NR	NR	Cox proportional hazards models were then used to determine the association of DDFS and BMI after adjusting for patient and tumor characteristics	Premenopausal, Postmenopausal	Lymphovascular invasion, systemic adjuvant treatment, and radiation therapy	39 mo	Patients with TN disease have a poor prognostic outcome regardless of BMI category. As such, the results of this study indicate that obesity does not function as a prognostic indicator among patients with TN disease.
25	Cleveland et al,^[45]^	USA	Prospective cohort follow-up of case-control study	N = 1508/Female	Mean = 62.1 (range, 2.7-88.6)	NR	NR	NR	NR	NR	NR	95% confidence interval both BMI < 25, BMI ≥ 25 Underweight = 607 Obese = 446	Pre-diagnosis recreational PA for ≥ 1 h/wk for ≥ 3 mo in any year over entire lifetime. PA assessed over time periods	NR	Adjustment for most factors did not substantially alter the estimates of effect	Premenopausal, Postmenopausal	chemotherapy, radiation, tamoxifen) for the original breast cancer diagnosis.	5.6 (range = 0.2–7.4) y	The association of Recreational physical activity (RPA) on breast cancer survival by other factors such as hormone receptor status, tumor stage, BMI and exogenous hormone use did not reveal any differential associations.
26	Haakinson et al,^[46]^	USA	Cohort study	N = 1352/Female	Mean = 66 (20–95)	LN status, Her2 status	NR	NR	NR	Stage–I = 1.252 (95% CI 0.668–2.345) Stage–II = 2.282 (95% CI 1.000–5.209) stage III = 1.343 (95% CI 0.259–6.950)	Genetic testing for BRCA1/2	95% confidence interval (CI). The range of BMI is < 30 kg/m^2^ to ≥ 30 kg/m^2^ Underweight = 1025 Obese = 327	The rates of locoregional recurrence were 2% for both obese and nonobese patients (*P* = .82). By Kaplan–Meier analysis.	NR	Obesity was found to be independently associated with mammographically detected tumors, larger tumor size, lymph node positivity, lower incidence of Her2 overexpression, and lower incidence of multifocality	Premenopausal, Postmenopausal	Surgical treatments, hormone therapy, chemotherapy, and radiotherapy	2.5 yr, with a range of 0–9.3 yr	Obese patients diagnosed with breast cancer are more likely to have their disease initially detected by imaging, have a larger tumor size, and have lymph node metastases. Although the treatment provided was similar to that provided to nonobese patients, obese patients trended toward worse overall survival
27	Lara-Medina et al,^[47]^	Mexico	Observational cohort study	N = 2074/Female	Median = 50 ± 12	HR-positive/HER2-negative, HER2-positive, and TNBC	NR	NR	NR	Stage I = 94.20 (88.91-99.49) II = 89.90 (84.81-94.99) III = 73.80 (67.53-80.07) IV = 33.80 (32.24-35.36)	test or the MannWhitney U test, Kolmogorov-Smirnov test. Pearson chi-square tests	95% confidence interval (CI), The range of BMI is < 25 kg/m^2^ to ≥ 30 kg/m^2^ Underweight = 1595 Obese = 479	NR	NR	Neoadjuvant chemotherapy with anthracyclines and taxanes.	Premenopausal, Postmenopausal	neoadjuvant chemotherapy	17 mo	Triple-negative breast cancer (TNBC) was associated with a higher risk of locoregional recurrence (LRR) and with lower disease-free survival (DFS) and cancer-specific survival (CSS) than those in patients with non-TNBC.
28	Ademuyiwa et al,^[48]^	Newyork	A retrospective study	N = 418/Female	Median = 54(range, 26–92)	Triple-negative breast cancers (TNBCs), Her-2/neu	NR	Underweight/Normal BMI = 124 (29.7%) Overweight BMI = 130 (31.1%) Obese BMI = 164 (39.2%)		Overall stage III –I = 4.42 (95% CI 2.45–7.99) Recurrence-Free stage III –I = 3.32(95% CI 1.94–5.67)	Wilcoxon rank-sum test and the Pearson chi-square test	95% confidence interval (CI), The range of BMI is 16.6 kg/m^2^ to 59.3 kg/m^2^ Underweight = 124 (29.7%) over weight = 130 (31.1%) Obese = 164 (39.2%)	NR	NR	triple negativity as any tumor that lacks expression of ER, PR, and Her-2/neu. ER and PR are measured using immunohistochemistry (IHC)	NR	adjuvant chemotherapy anthracycline-based regimen (80%); doxorubicin plus cyclophosphamide followed by a taxane	37.2 months	No significant relation between obesity and RFS or OS emerged in patients with TNBC after controlling for clinically significant factors
29	Dal Maso et al,^[49]^	Italy	Multicenter hospital-based case–control study	N = 1453/Female	Median = 55 (23–74)	NR	NR	NR	NR	Stage–I = 1 Stage–II = 2.52 (95% CI 1.88–3.37) stage III–IV = 6.17 (95% CI 4.48–8.50)	NR	95% confidence interval (CI). The range of BMI is < 25 kg/m^2^ to ≥ 30 kg/m^2^ Underweight = 814 over weight = 464 Obese = 172	Occupational physical activity at age 30 years and leisure time physical activity were not significantly associated to all cause or breast cancer mortality	Smoking could conceivably decrease the risk of breast cancer death by promoting a lower body mass index	Region of residence, age at diagnosis, year at diagnosis, TNM stage, ER/PR status	Premenopausal, Postmenopausal	NR	12.6 yr	No significant relationship with survival after breast cancer emerged for several other major lifestyle factors, including physical activity. High BMI was the lifestyle risk factor that most consistently modified breast cancer prognosis.
30	Chow et al,^[50]^	China	Population-based case control study	N = 198/Female	Mean = 55.4	NR	NR	NR	NR	NR	NR	95% confidence interval (CI). The range of BMI is < 19 to > 31 kg/m^2^	NR	increased risk of breast cancer, respectively, after adjustment for non-anthropometric risk factors	The ORs (Odds ratios) for breast cancer increased with increasing BMI (> 23, 1.73; > 27, 2.06; > 31, 3.82 after adjusting for nonanthropometric risk factors)	Premenopausal, Postmenopausal	NR	NR	Weight control in obese women may be an effective measure for breast cancer prevention in postmenopausal women
31	Ghiasvand et al,^[51]^	Iran	Case control study	N = 521/Female	Mean = 41.24	NR	\<152 = 112 (22.4) 153–157 = 154 (30.8) 158–160 = 98 (19.6) >160 = 135 (27.2)	NR	NR	NR	Mann–Whitney *U* test	95% confidence interval (CI). The range of BMI is < 25 to > 30 kg/m^2^ Underweight = 168 (34.0) Normal weight = 195 (39.4) Obese = 132 (26.6)	NR	NR	After adjustment in the logistic model only occupation, parity, menopausal status, OC usage, family history of breast cancer and breast feeding revealed significant associations with the risk of breast cancer	Premenopausal, Postmenopausal	Women with history of hysterectomy or oophorectomy	NR	Logistic regression performed to investigate associations of reproductive and anthropometric factors with breast cancer risk
32	Lotfi et al,^[52]^	Iran	Case-control study	N = 160/Female	Mean = 48.9 ± 9.7	NR	NR	NR	NR	NR	NR	95% confidence interval (CI). The range of BMI is < 25 to > 30 kg/m^2^ Underweight = 17 (22.4) Normal weight = 22 (28.9) Obese = 37 (48.7)	History of not having regular leisure time physical activity and history of exposure to X-ray up to 30 years	NR	NR	NR	NR	NR	Positive familial history of breast cancer amongst the first grades, early onset of the first menstruation cycle and less duration of breastfeeding were significantly associated with breast cancer
33	Montazeri et al,^[53]^	Iran	Case-control study	N = 116/Female	Mean = 54.6	NR	≤ 157 = 61 (53) 158–160 = 29 (25) 161–164 = 19 (16) ≥ 165 = 7 (6)	NR	NR	NR	Logistic regression, T–test, Chi-square	95% confidence interval (CI). The range of BMI is < 18 to > 30 kg/m^2^ Underweight = 23 (20) Normal weight = 51 (44) Obese = 42 (36)	NR	NR	NR	Premenopausal, Postmenopausal	NR	NR	Women with a BMI in the obese range had a 3-fold increased risk of breast cancer
34	Ghiasvand et al,^[54]^	Iran	Case-control study	N = 493/Female	Median = 56	NR	≤ 151 = 143 152–156 = 160 157–160 = 90 > 160 = 85	NR	NR	NR	Wald test statistics.	95% confidence interval (CI). The range of BMI is < 18 to > 30 kg/m^2^ Underweight = 4 Normal = 78 Overweight = 126 Obese = 89	Physical activity due to traditional way of life, early life events and conditions may play a role for the breast cancer risk in parous women	NR	Logistic regression with adjustment for the matching factors to estimate the odds ratios (ORs) and 95% confidence intervals (CI).	Premenopausal, Postmenopausal	NR	NR	The family history was significantly associated with increased breast cancer risk, but not increasing height, early age at menarche, late age at first birth or short breastfeeding.
35	Sepandi et al,^[55]^	Iran	Case-control study	N = 11,850/Female	Median = 49.4	NR	<152 = 54 (31) 153-159 = 86 (59.3) >160 = 14 (9.7)	NR	NR	NR	NR	95% confidence interval (CI). The range of BMI is < 25 to > 30 kg/m^2^ Underweight = 58 (29.6) Normal = 81 (41.3) Obese = 57 (29.1)	NR	NR	After adjustment in the logistic model, only occupation, parity, menopausal status, OC usage, family history of breast cancer, and breastfeeding revealed significant associations with the risk of breast cancer	Premenopausal, Postmenopausal	NR	NR	Logistic regression was performed to investigate associations of reproductive and anthropometric factors with breast cancer risk.
36	Zare et al,^[56]^	Iran	Case-control study	N = 25,592/Female	Median = 49.18 ± 8.86	NR	NR	NR	NR	NR	NR	95% confidence interval (CI). The range of BMI is < 25 to > 30 kg/m^2^ Underweight = 21 (0.37) Normal = 41 (0.57 Obese = 23 (0.37)	NR	NR	NR	Premenopausal, Postmenopausal	NR	NR	The multivariate model revealed that the past history of ovarian cancer, hormone therapy, and first relatives with breast cancer were associated with increased risk for breast cancer
37	Abbasi et al,^[57]^	Malaysia	Case-control study	N = 150/Female	Median = 47.49 ± 11.43	NR	NR	NR	NR	Stage II = 133 (88.7%) Stage III = 15 (10.0%) Stage IV = 2 (1.3%)	χ2 tests	95% confidence interval (CI). The range of BMI is < 18 to > 30 kg/m^2^ Underweight = 5 (3.3%) Normal = 57 (38.0%) Overweight = 55 (36.7%) Obese = 33 (22.0%)	NR	NR	NR	Premenopausal, Postmenopausal	NR	NR	In multivanate analysis, only body mass index or BMI age at menarcne, age at marriage, race, ABO and Rh blood groups and family history of breast cancer were associated with significantly increased risk for breast cancer
38	Dianatinasab et al,^[58]^	Iran	Case-control study	N = 1052/Female	Mean = 47.8	NR	NR	NR	NR	NR	Chi-square, independent sample t-test	95% confidence interval (CI). The range of BMI is < 18 to > 30 kg/m^2^ Underweight = 34 (6.5%) Normal = 194 (36.9%) Overweight = 169 (32.1%) Obese = 93 (17.7%)	Stress and hair coloring and to some extend physical inactivity seem to expose women to higher risk of BC than other factors under study	NR	The alpha value and power were set at 0.05 (two sided) and 80% respectively. Univariate analyses (Chi-square, independent sample t-test) was conducted to calculate unadjusted associations	Premenopausal, Postmenopausal	NR	NR	Marital age, age at first delivery, birth interval, life time breast feeding, and menarche age as significant associates of breast cancer.
39	Ekpanyaskul et al,^[59]^	Thailand	Case-control study	N = 516/Female	Mean = 47.8 ± 10.0	NR	NR	277 (53.68%)	227 (43.99%)	NR	Chi-square test	95% confidence interval (CI). The range of BMI is < 18 to > 25 kg/m^2^ Underweight = 338 (65.50%) Normal = 37 (7.17%) Overweight = 141 (27.33%)	NR	NR	After adjusting for confounding factors by multiple logistic regression analysis, the results showed that occupational category as production and related workers	Premenopausal, Postmenopausal	NR	NR	Occupational category as production and related workers, transport equipment operators and laborers was associated with an increased risk of breast cancer
40	Gao et al,^[60]^	Singapore	Case-control study	N = 28,883/Female	Mean= >/85	NR	NR	NR	NR	NR	NR	95% confidence interval (CI). The range of BMI is < 23 to > 27 kg/m^2^ Underweight = 476) Normal = 497 Overweight = 223	NR	NR	NR	NR	NR	5-yr	Age-atmenarche, age-at-birth of first live child and number of first-degree-relatives performed similarly with associated concordance statistics
41	Jordan et al,^[61]^	Thailand	Case-control study	N = 47,271/Female	Median = 39 (28 to 51)	NR	154 cm = 370 (44) 155–159 cm = 215(25) 160 cm = 262 (31)	NR	NR	NR	NR	95% confidence interval (CI). The range of BMI is < 18 to > 25 kg/m^2^ Underweight = 47 (6%) Normal = 475 (56%) Overweight = 162 (19%) Obese = 162 (19%)	Physical activity decreases the risk of post-menopausal breast cancer only	NR	NR	NR	NR	NR	Substantial increases in breast cancer rates in Thailand could be expected in the future
42	Marzbani et al,^[62]^	Iran	Case-control study	N = 620/Female	Mean = 41.5 ± 6.2	NR	NR	NR	NR	NR	NR	95% confidence interval (CI). The range of BMI is < 24 to > 34 kg/m^2^ Underweight = 140 Normal = 166 Overweight = 84 Obese = 18	NR	NR	NR	NR	NR	2 yr	A dose-response model indicated that increasing vegetable and fruit consumption up to 90 servings per month decreased the odds of breast cancer
43	Mobarakeh et al,^[63]^	Iran	Case-control study	N = 93/Female	Mean = 20–65	NR	NR	NR	NR	NR	Student *t* test; Fishers’ Exact Test; Chi-Square Test; NPar Tests (Mann-Whitney);	95% confidence interval (CI). The range of BMI is < 25 to > 29 kg/m^2^ Normal = 64.91(11.81%) Overweight = 24.89 (4.54%)	NR	NR	The risk of cancer was analyzed after adjustment for confounding factors. Age, weight, body mass index (BMI), waist circumference, educational status, parity, lactation, marital status, menopause, history of estrogen therapy	Menopausal status	NR	NR	High intake of fat dairy products including milk and cheese was found to be a statistically significant factor for increasing breast cancer risk in models
44	Sangrajrang et al,^[64]^	Thailand	Case-control study	N = 570/Female	Mean = 43.2 ± 12.4	NR	NR	316 (63.7)	180 (36.3)	NR	Hardy–Weinberg equilibrium, Fisher’s exact test, Cochran–Armitage trend test	95% confidence interval (CI). The range of BMI is < 20 to > 25 kg/m^2^ Underweight = 111 Normal = 269 Overweight = 117	NR	NR	The adjusted ORs and 95% CIs for OR were calculated by the multivariate logistic regression analyses	Premenopausal, Postmenopausal	NR	6 mo	A stratified analysis by menopausal status indicated the association between the NAT2 SNP and breast cancer was mainly evident in premenopausal women
45	Sangrajrang et al,^[65]^	Thailand	Case-control study	N = 1142/Female	Mean = 43.7 ± 11.6	NR	NR	749 (65.7%)	390 (34.3%)	NR	NR	95% confidence interval (CI). The range of BMI is < 20 to > 25 kg/m^2^ Underweight = 712 Normal = 95 Overweight = 329	Demographic and anthropometric data, reproductive and medical history, residential history, physical activity and occupation as well as dietary habits	NR	NR	Premenopausal, Postmenopausal	NR	NR	Regular exercise was associated with a decreased risk of breast cancer
46	Tan et al,^[66]^	Malaysia	Case-control study	N = 3980/Female	Mean = 54.0 and 50.8	NR	<1.53 = 399 1.53 ± 1.57 = 666 >1.57 = 950	1550	2408	NR	Chi-square teasts	95% confidence interval (CI). The range of BMI is < 23 to > 27 kg/m^2^ Underweight = 757 Normal = 758 Overweight = 493	NR	NR	BMI, which was associated with a lower risk of breast cancer after adjustment for major breast cancer risk factors.	Premenopausal, Postmenopausal	NR	NR	Ever breastfed, longer breastfeeding duration, a higher soymilk and soy product intake, and a higher level of physical activity were associated with lower risk of breast cancer

NR = not reported.

### 3.3. Risk of bias

The Newcastle–Ottawa checklist was used to risk of bias assessment of the included studies. The risk of bias assessment for each study is shown in Table 2. Among the assessed 46 studies, 33 studies have a “moderate risk,” whereas the remaining 13 studies have a “low risk.” No study was considered to have a “high risk” of bias.

**Table 2:** Risk of bias assessment the included studies.

S. No	Study of Author and year	Selection				Comparability	Outcome			Overall score	Risk of Bias
		Exposed representation b	Non-exposed selection b	Ascertainment of obesityc	Outcome absent at study start d	Adjustment by age and nodal status or stage e	Outcome assessment b	Follow-up length f	Adequacy of follow-up g		
1	Smith et al,^[21]^	Y	Y	Y	Y	—	Y	Y	—	6	Moderate
2	Saleh et al,^[22]^	Y	Y	Y	Y	—	Y	Y	—	6	Moderate
3	Abubakar et al,^[23]^	Y	Y	Y	Y	Y	Y	—	—	6	Moderate
4	Gondo et al,^[25]^	Y	Y	—	Y	Y	Y	Y	—	6	Moderate
5	Krasniqi et al,^[24]^	Y	Y	—	Y	Y	Y	Y	—	6	Moderate
6	Ayoub et al,^[26]^	Y	Y	Y	Y	Y	Y	—	—	6	Moderate
7	Cho et al,^[27]^	Y	Y	Y	Y	Y	Y	—	—	6	Moderate
8	Engmann et al,^[29]^	Y	Y	Y	Y	Y	Y	—	—	6	Moderate
9	Al Jarroudi et al, m^[28]^	Y	Y	Y	Y	Y	Y	—	—	6	Moderate
10	Bao et al,^[30]^	Y	Y	Y	Y	Y	Y	Y	Y	8	Low
11	Chen et al,^[31]^	Y	Y	Y	Y	—	Y	—	Y	6	Moderate
12	Kawai et al,^[32]^	Y	Y	Y	Y	Y	Y	Y	—	7	Low
13	Cecchini et al,^[33]^	Y	Y	Y	Y	Y	Y	Y	—	7	Low
14	Jeon et al,^[34]^	Y	Y	Y	Y	Y	Y	Y	—	7	Low
15	Widschwendter et al,^[35]^	Y	Y	Y	Y	Y	Y	Y	—	7	Low
16	Ohara et al,^[36]^	Y	Y	Y	Y	—	Y	—	Y	6	Moderate
17	Robinson et al,^[37]^	Y	Y	—	Y	Y	Y	Y	—	6	Moderate
18	Asaga et al,^[38]^	Y	Y	Y	Y	—	Y	—	Y	6	Moderate
19	Crozier et al,^[39]^	Y	Y	Y	Y	Y	Y	Y	Y	8	Low
20	Mazzarella et al,^[40]^	Y	Y	Y	Y	Y	Y	Y	Y	8	Low
21	Turkoz et al,^[41]^	Y	Y	Y	Y	Y	Y	—	—	6	Moderate
22	Biglia et al,^[42]^	Y	Y	Y	Y	Y	Y	Y	—	7	Low
23	Schmidt et al,^[43]^	Y	Y	Y	Y	—	Y	Y	Y	7	Low
24	Dawood et al,^[44]^	Y	Y	Y	Y	Y	Y	—	—	6	Moderate
25	Cleveland et al,^[45]^	Y	Y	Y	Y	—	Y	Y	Y	7	Low
26	Haakinson et al,^[46]^	Y	Y	Y	Y	Y	Y	Y	Y	8	Low
27	Lara-Medina et al,^[47]^	Y	Y	Y	Y	—	Y	—	—	5	Moderate
28	Ademuyiwa et al,^[48]^	Y	Y	Y	Y	Y	Y	—	—	6	Moderate
29	Dal Maso et al,^[49]^	Y	Y	Y	Y	Y	Y	Y	Y	8	Low
30	Chow et al,^[50]^	Y	Y	Y	Y	Y	Y	—	—	6	Moderate
31	Ghiasvand et al,^[51]^	Y	—	Y	Y	—	Y	—	—	4	Moderate
32	Lotfi et al,^[52]^	Y	Y	Y	Y	Y	Y	—	—	6	Moderate
33	Montazeri et al,^[53]^	Y	Y	Y	Y	Y	Y	—	—	6	Moderate
34	Ghiasvand et al,^[54]^	Y	Y	Y	Y	Y	Y	—	—	6	Moderate
35	Sepandi et al,^[55]^	Y	Y	—	Y	—	Y	—	—	4	Moderate
36	Zare et al,^[56]^	Y	—	Y	Y	Y	Y	—	—	5	Moderate
37	Abbasi et al,^[57]^	Y	Y	Y	Y	—	Y	—	—	5	Moderate
38	Dianatinasab et al,^[58]^	Y	—	Y	Y	Y	Y	—	—	5	Moderate
39	Ekpanyaskul et al,^[59]^	Y	Y	—	Y	Y	Y	—	—	5	Moderate
40	Gao et al,^[60]^	Y	Y	Y	Y	Y	Y	—	—	6	Moderate
41	Jordan et al,^[61]^	Y	Y	Y	Y	Y	Y	—	—	6	Moderate
42	Marzbani et al,^[62]^	Y	—	Y	Y	—	Y	Y	—	5	Moderate
43	Mobarakeh et al,^[63]^	Y	Y	—	Y	Y	Y	—	—	5	Moderate
44	Sangrajrang et al,^[64]^	Y	Y	Y	Y	Y	Y	Y	—	7	Low
45	Sangrajrang et al,^[65]^	Y	Y	Y	Y	—	Y	—	—	5	Moderate
46	Tan et al,^[66]^	Y	Y	Y	Y	Y	Y	—	—	6	Moderate

### 3.4. Clinical characteristics

Among included 46 studies in this review, 50% (n = 23) of the studies found the HER2 type of breast cancer followed by triple-negative and HR-positive. Most of the included studies were included both premenopausal and postmenopausal status. The follow-up duration was ranging from 6 months to 16.2 years. The findings of the included studies were demonstrated that the higher BMI is associated with greater breast cancer risk.

### 3.5. Impact of BMI on breast cancer

The meta-analysis comprised 16 case-control studies in total. Among the selected 16 studies, the BMI data was calculated into 2 groups, including overweight (BMI > 25 to < 30 kg/m^2^) and Obese (BMI ≥ 30). The OR was used in selected studies to examine the effect of a high BMI (overweightness and obesity) on breast cancer. The random effects approach was used to analyses studies, which revealed that they were heterogeneous.

### 3.6. Obesity

**Figure 2.**
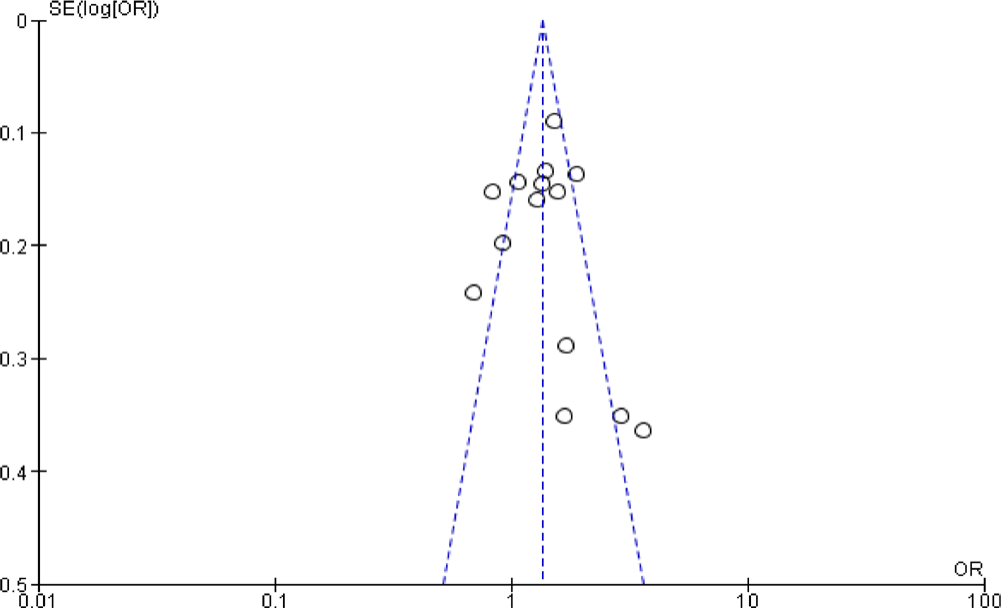
Funnel plot of odds ratio estimates of breast cancer by obesity.

**Figure 3.**
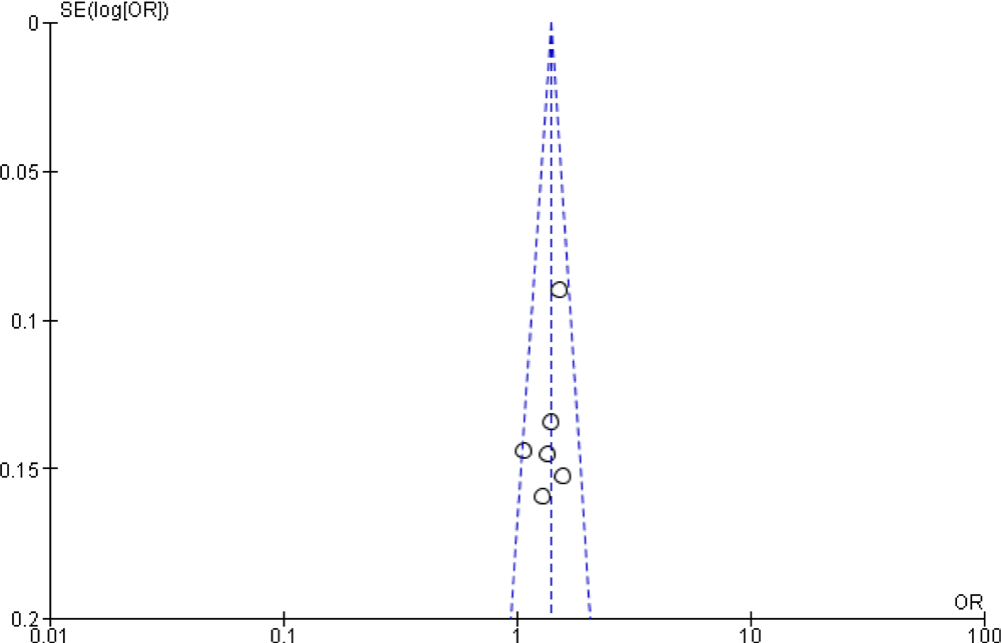
Forest plot of odds ratio estimates of breast cancer by obesity.

Fourteen studies report the Meta analysis between case and control group for obesity (BMI ≥ 30 kg/m^2^). The study reported a significant difference between the case and control groups (*P* < .001). The study reported that obesity was significantly higher in the case group compared with the control group (*P* < .001). Heterogeneity between the 14 studies is medium (*I*^2^ = 72%). Test for overall effect: Z = 3.55 (*P* = .0004) (OR = 1.37 CI: 1.15–1.63) (Table 3, Figs. 2 and 3).

**Table 3:** Results for the Categorical analysis of the association between BMI and breast cancer after removing the studies which lies outside the funnel.

Author/study	OR (Odds ratio)	
	Overweight (95% CI)	Obese (95% CI)
Meta-analysis: OR		
Abbasi et al,^[57]^	1.85 [1.12, 3.07]	No data
Ekpanyaskul et al,^[59]^	1.68 [1.29, 2.19]	No data
Dianatinasab et al,^[58]^	No data	1.57 [1.16, 2.11]
Gao et al,^[60]^	1.25 [1.01, 1.56]	1.39 [1.07, 1.81]
Ghiasvad.,^[51]^	1.02 [0.77, 1.35]	1.07 [0.80, 1.41]
Ghiasvad^[54]^	1.26 [0.95, 1.67]	1.35 [1.01, 1.79]
Lotfi et al,^[52]^	0.80 [0.40, 1.58]	No data
Marzbani et al,^[62]^	1.28 [0.91, 1.78]	No data
Montazeri et al,^[53]^	1.70 [0.97, 3.00]	No data
Sangrajrang et al,^[65]^	No data	1.52 [1.28, 1.82]
Sepandi et al,^[55]^	1.29 [0.95, 1.76]	1.29 [0.95, 1.77]
Zare et al,^[56]^	0.87 [0.55, 1.38]	No data

### 3.7. Overweight

**Figure 4.**
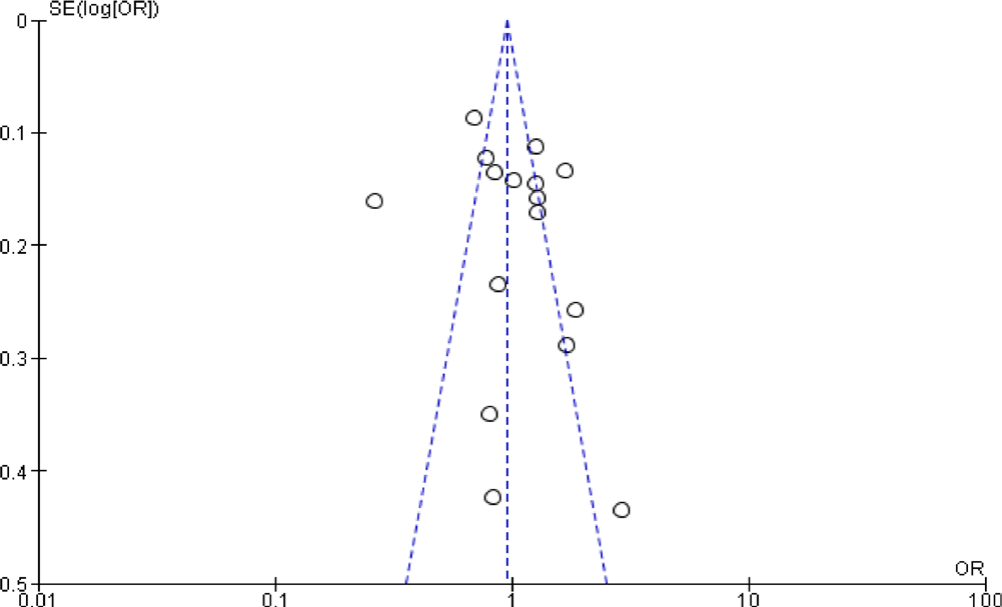
Funnel plot of odds ratio estimates of breast cancer by obesity after removing studies which lies outside the funnel.

**Figure 5.**
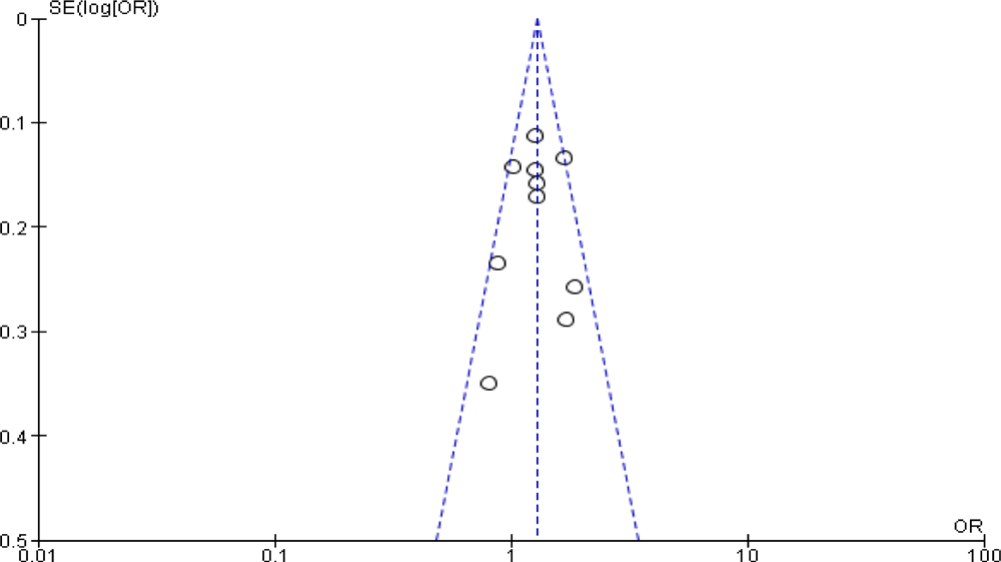
forest plot of odds ratio estimates of breast cancer by obesity after removing studies which lies outside the funnel.

Sixteen studies report the Meta analysis between case and control group for overweight (25 < BMI < 30 kg/m^2^). The study reported no significant relation between overweightness and breast cancer in women (*P* > .05). Heterogeneity between the 14 studies is medium (*I*^2^ = 89%). Test for overall effect: Z = 1.47 (*P* = .77 > 0.05) (OR = 0.95 CI: 0.88–1.02) (Table 3, Figs. 4 and 5). The risk of being overweight was determined to be Chi^2^ = 134.56, *P* = .77, *I*^2^ = 89%, and the difference across trials was Tau^2^ = 0.20.

### 3.8. Sub-group analysis

**Figure 6.**
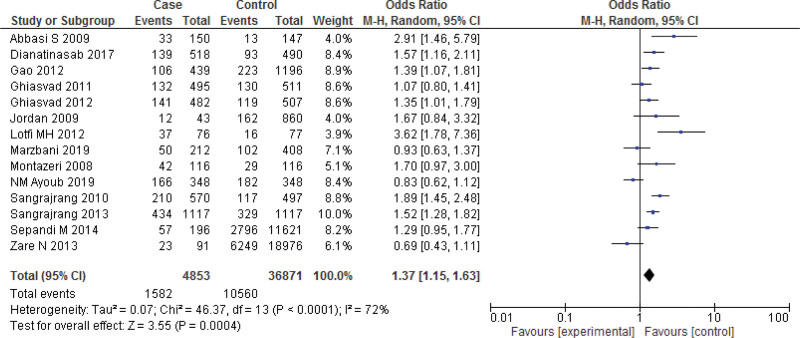
Funnel plot of odds ratio estimates of breast cancer by overweight.

**Figure 7.**
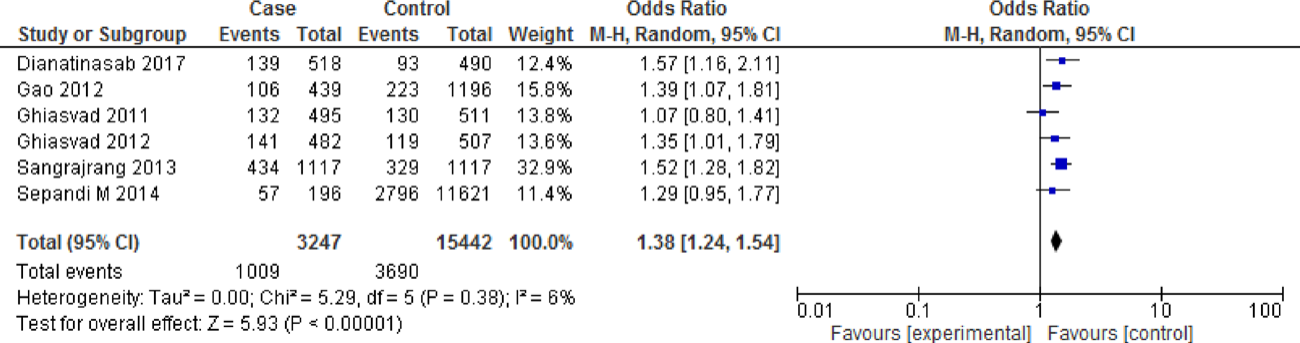
Forest plot of odds ratio estimates of breast cancer by overweight.

Eight studies^[26,52,53,56,57,61,62,65]^ lies outside the funnel, so remove the studies and rerunning the analysis we get 6 studies reports the Meta analysis between case and control group for obese (BMI ≥ 30 kg/m^2^). The study reported a significant difference between the case group and control group (*P* < .001). Obesity was found to be significantly higher in the case group than in the control group (*P* < .001). Heterogeneity between the 6 studies is low (*I*^2^ = 6%). Test for overall effect: Z = 5.93 (*P* < .0001) (OR = 1.38 CI: 1.24–1.54) (Figs. 6 and 7). The risk of obesity was found to be Chi^2^ = 5.29, *P* < .0001, *I*^2^ = 6%, and the difference across trials was Tau^2^ = 0.20.

**Figure 8.**
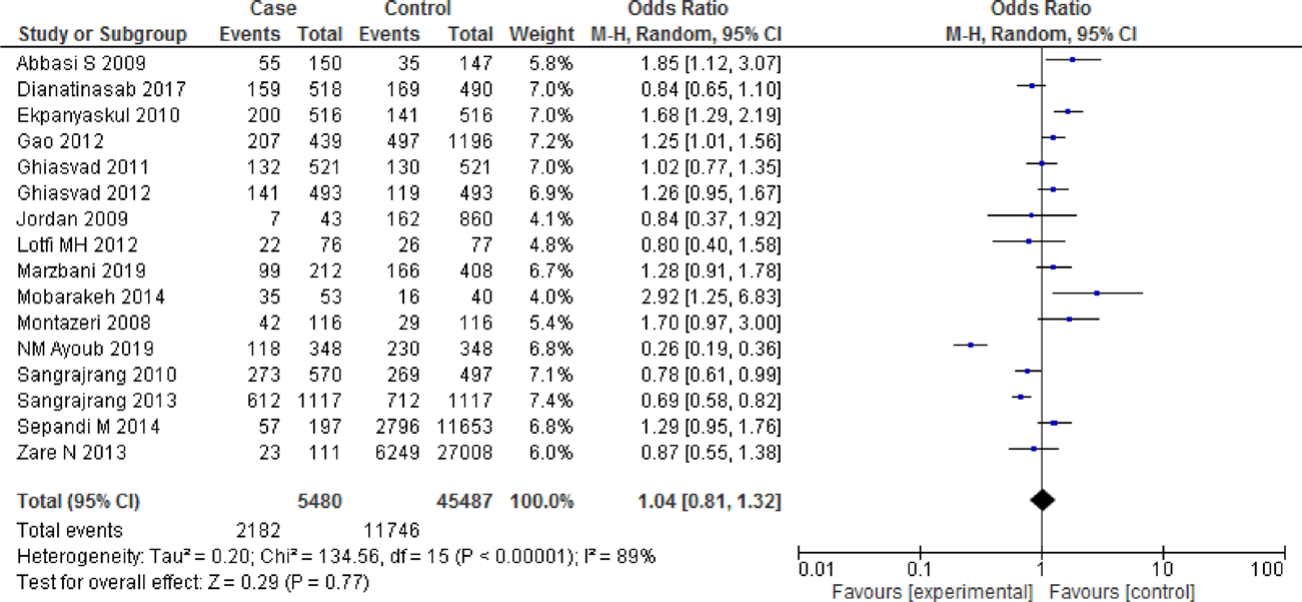
Funnel plot of odds ratio estimates of breast cancer by overweight after removing studies which lies outside the funnel.

**Figure 9.**
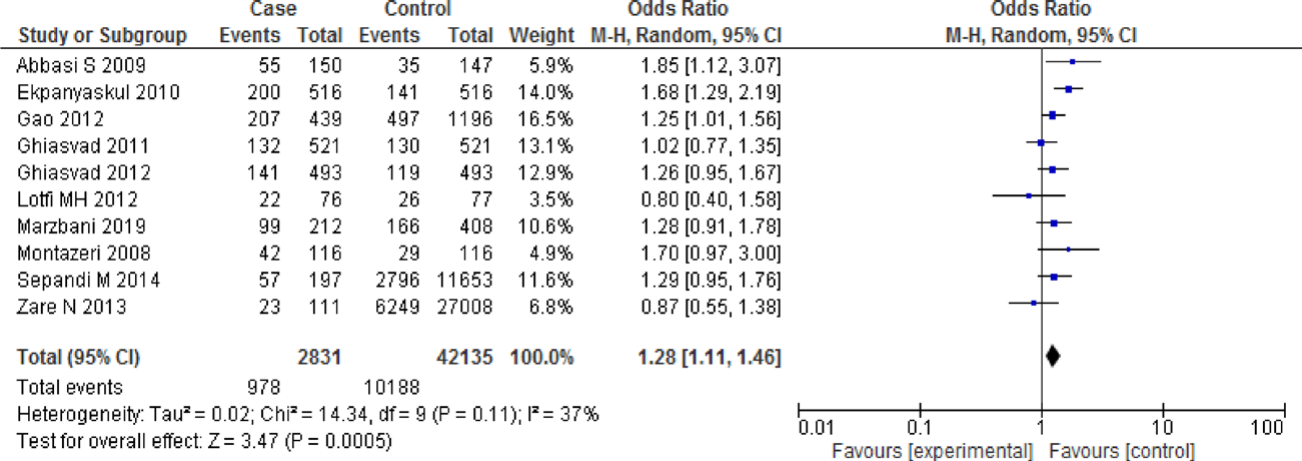
Forest plot of odds ratio estimates of breast cancer by overweight after removing studies which lies outside the funnel.

Six studies^[26,58,61,63,64,65]^ lies outside the funnel, so remove the studies and rerunning the analysis we get 10 studies reports the Meta analysis between case and control group for overweight (25 < BMI < 30 kg/m^2^). The study reported a significant relation between overweightness and breast cancer in women (*P* = .0005). Heterogeneity between the 14 studies is medium (*I*^2^ = 37%). Test for overall effect: Z = 3.47 (*P* = .0005) (OR = 1.28 CI: 1.11–1.46) (Figs. 8 and 9). The risk of being overweight was found to be Chi^2^ = 14.34, *P* = .0005, *I*^2^ = 37%, and the difference across studies was Tau^2^ = 0.02.

## 4. Discussion

Obesity is a worldwide epidemic, with 69% of people in the United States and 38% worldwide being overweight or obese.^[67]^ Obesity is linked to changes in whole-body physiology and hormonal environment, which can lead to a variety of diseases. Obesity has been linked to a poor prognosis in a variety of cancers, including breast cancer.^[68]^ BMI has been used as a marker of generalized obesity in the majority of research and meta-analyses available and found an elevated risk of breast cancer in obese or overweight women of all ethnic groups during the postmenopausal period.^[51,68]^ In this study, we review the evidence of the relationship between BMI and breast cancer, with an emphasis on breast cancer prognosis.

In this review, 46 included studies comprising 2,95,260 patients were included to explore the relationship between breast cancer risk and BMI. Obesity was significantly higher in the case group compared to the control group (*P* < .001), according to the 16 case-control studies included in the meta-analysis. Heterogeneity between the 14 studies is medium (*I*^2^ = 72%). After removing the publication bias a significant difference between the case group and control group (*P* < .001) for obesity was observed. A study examining breast cancer risk factors in the Eastern Mediterranean region (EMRO) by Namiranian et al showed that being obesity increases the breast cancer incidence, and this association was statistically significant,^[69]^ confirming the current findings. Furthermore, Jee et al^[70]^ discovered that a higher BMI might raise the incidence of breast cancer in the Korean population. In this review, there was no significant relation between overweightness and breast cancer in women (*P* > .05). Heterogeneity between the 14 studies is medium (*I*^2^ = 89%). However, after removing the publication bias a significant relation between overweightness and breast cancer in women (*P* = .0005) was observed. Similarly, in a review by Zahmatkesh et al,^[3]^ estimate the odds ratio of obesity and overweight as risk factors for breast cancer. Obesity was discovered to have a strong link to the breast cancer risk (OR = 1.81, 95% CI = 1.24–2.64). There was also a link found between being overweight and the risk of breast cancer (OR = 1.46, 95% CI = 1.13–1.89).

Although the specific mechanism behind the association between BMI and breast cancer risk is uncertain, numerous possibilities have been proposed. The higher level of estrogen produced by the aromatization of and rostenedione in the larger fat reserves of postmenopausal women was assumed to be the reason of the positive relationship between BMI and breast cancer risk.^[7]^ The protective impact of greater weight in the early premenopausal years, it is linked to longer anovulatory cycles and decreased progesterone and estrogen levels, is likely to have contributed to the negative link between higher BMI and breast cancer risk in premenopausal women. However, more investigations of carcinogenic pathways will be required to confirm these predictions. The evaluation of study quality, the extensive literature search process, and the large number of studies included are all strengths of our review. The investigations were conducted in a range of places throughout the world, with a diverse breast population adding to the data’ generalizability. However, the present review has some limitations. Firstly, we used subgroup analysis to find the major source of heterogeneity after observing it across studies. Secondly, the meta-analysis included only case-control studies. Thirdly, our meta-analysis’ findings were confined to Iran, Thailand, Malaysia, and Singapore, and may not be applicable to other locations such as Africa or the United States. Despite these limitations, this updated review provides an evidence-based report on the impact of BMI on breast cancer demonstrated by pooled effect of different studies using rigours methodology.

## 5. Conclusion

Obese women with breast cancer are a specific type of patient. Women with higher BMI are more likely to develop cancer as well as increased surgery and radiation difficulties. Obesity and overweight in women greatly increase the risk of breast cancer, according to the findings of the current meta-analysis. More research is needed to confirm these findings and understand the pathogenic pathways.

## Author contributions

**Formal analysis:** Mohamed Chahine.

**Writing – original draft:** Nikolaos Tzenios.

**Writing – review & editing:** Mary E. Tazanios.
